# The association of dietary nutrients consumption with hepatic steatosis and fibrosis from NHANES 2017–2020

**DOI:** 10.3389/fnut.2025.1510860

**Published:** 2025-06-24

**Authors:** Qi Sheng, Shousheng Liu, Haiyang Yu, Huanchen Shi, Yongning Xin

**Affiliations:** ^1^Department of Infectious Disease, Qingdao Municipal Hospital, Shandong University, Jinan, China; ^2^Department of Clinical Nutrition, The Affiliated Hospital of Qingdao University, Qingdao, China; ^3^Department of Gastroenterology, Qingdao Municipal Hospital, Shandong University, Jinan, China; ^4^Clinical Research Center, Qingdao Municipal Hospital, Qingdao, China; ^5^Department of Radiology, The Affiliated Hospital of Qingdao University, Qingdao, China; ^6^Department of Liver Surgery and Transplantation, Liver Cancer Institute and Zhongshan Hospital, Fudan University, Shanghai, China

**Keywords:** daily nutrients consumption, hepatic steatosis, fibrosis, National Health and Nutrition Examination Survey, chronic liver disease

## Abstract

**Background/objectives:**

Hepatic steatosis and fibrosis represent significant and growing global health burdens. There is an urgent need to seek strategies for early prevention and control of hepatic steatosis and fibrosis. This study attempted to comprehensively evaluate the relationship between dietary nutrient intake and the risk of hepatic steatosis and fibrosis to provide assistance for doctors in guiding the diet of the patients.

**Methods:**

This observational study assembled 15,560 participants from the 2017–March 2020 cohorts of the National Health and Nutrition Examination Survey. 34 nutrient intake items were included. The liver ultrasound transient elastography was used to evaluate hepatic steatosis and hepatic fibrosis. Various variables, encompassing sociodemographic characteristics, and other potential confounders were considered to ensure the stability of the findings. Additionally, the analysis accounted for various covariates and employed restricted cubic spline analysis to examine potential nonlinear relationships. Weighted quantile sum (WQS) (mixed effect) models were used in the analysis.

**Results:**

The negative correlations were found between low carbohydrate, vitamin C, pyridoxine, magnesium, iron and potassium intake with controlled attenuation parameter (CAP) after adjusting all the covariates and excluding non-linear correlations. Nonlinear correlation was found to exist between the consumption of energy, vitamin E, folate, sodium, alcohol, *α*-Linolenic acid and fish oil and hepatic steatosis (*p* < 0.05). The negative correlations were showed between low dietary fiber per energy and phosphorous intake with liver stiffness measurement (LSM) after adjusting all the covariates and excluding non-linear correlations (*p* < 0.05). High caffeine intake showed the positive correlation with LSM in Model3 after adjusting all covariates (*p* = 0.022). The majority of dietary nutrients intake were found to have nonlinear relationships with liver fibrosis.

**Conclusion:**

Overall, many nutrient variables were newly identified associations with hepatic steatosis and fibrosis. Critical threshold intake levels were revealed that may elevate disease risk. These findings may help us better understand the complex relationship between diet and hepatic steatosis and fibrosis. Moreover, this data provides critical insights for establishing evidence-based clinical nutrition strategies to optimize the prevention and management of liver diseases.

## 1 Introduction

Chronic liver disease has emerged as a major public health burden worldwide, which can develop into cirrhosis and liver failure (1, 2), diminishing quality of life and even resulting in death (3, 4). Hepatic steatosis and fibrosis are common pathophysiological process during the development of liver diseases (5). Hepatic steatosis is a condition that occurs when excess fat builds up in the liver. It can be caused by a number of factors, including an imbalance of lipids, insulin resistance, and metabolic syndrome. Hepatic steatosis is increasingly common and represents a very frequent diagnosis in the medical field, which could contributes to the progression toward liver fibrosis (6). Despite the use of limited herbal medicines (e.g., silymarin, quercetin, hesperidin, and berberine) and natural compounds with fewer side effects in preventing and treating liver diseases, there is still a lack of effective treatment methods for hepatic steatosis and fibrosis (7–9).

Liver ultrasound transient elastography is widely used to provide objective measures for hepatic steatosis and fibrosis. By measuring the velocity of the mechanically generated shear wave through the liver, the transient elastography has been found to better diagnostic accuracy than other non-invasive diagnostic methods (10–12). Controlled attenuation parameter (CAP) and liver stiffness measurement (LSM) were obtained to assess hepatic steatosis and fibrosis (13).

Nutrition factors were revealed to have significant impacts on hepatic steatosis and fibrosis (14–17). However, there is a lack of large-scale cohort study research on the comprehensive analysis of the correlation between various nutrients and hepatic steatosis/fibrosis. The NHANES is the most in-depth survey that measures the health and nutrition of the US national population. Current research landscapes reveal persistent gaps in systematically elucidating the pan-nutrient hepatoprotective mechanisms through large-scale cohort studies, particularly regarding dose-dependent effects across liver disease spectra16. Notably, even for extensively investigated micronutrients (e.g., iron overload in steatosis, vitamin D pleiotropy), conflicting evidence persists about their therapeutic thresholds and pathophysiological duality (protective vs. pro-fibrotic roles). This survey collected data through interviews, standard exams, and biospecimen collection, providing a standardized environment for the health examinations (18). Liver ultrasound transient elastography was conducted for the first time from 2017 (19), while the latest updated data of the results is up to March 2020. We wanted to use the latest data from the cycles of the 2017–March 2020 to explore the associations between dietary nutrition consumption with hepatic steatosis and fibrosis. Therefore, in order to explore the relationship between nutrition and chronic liver disease, we conducted from the perspective of dietary nutrition intake.

## 2 Materials and methods

### 2.1 Study population

**Figure 1:**
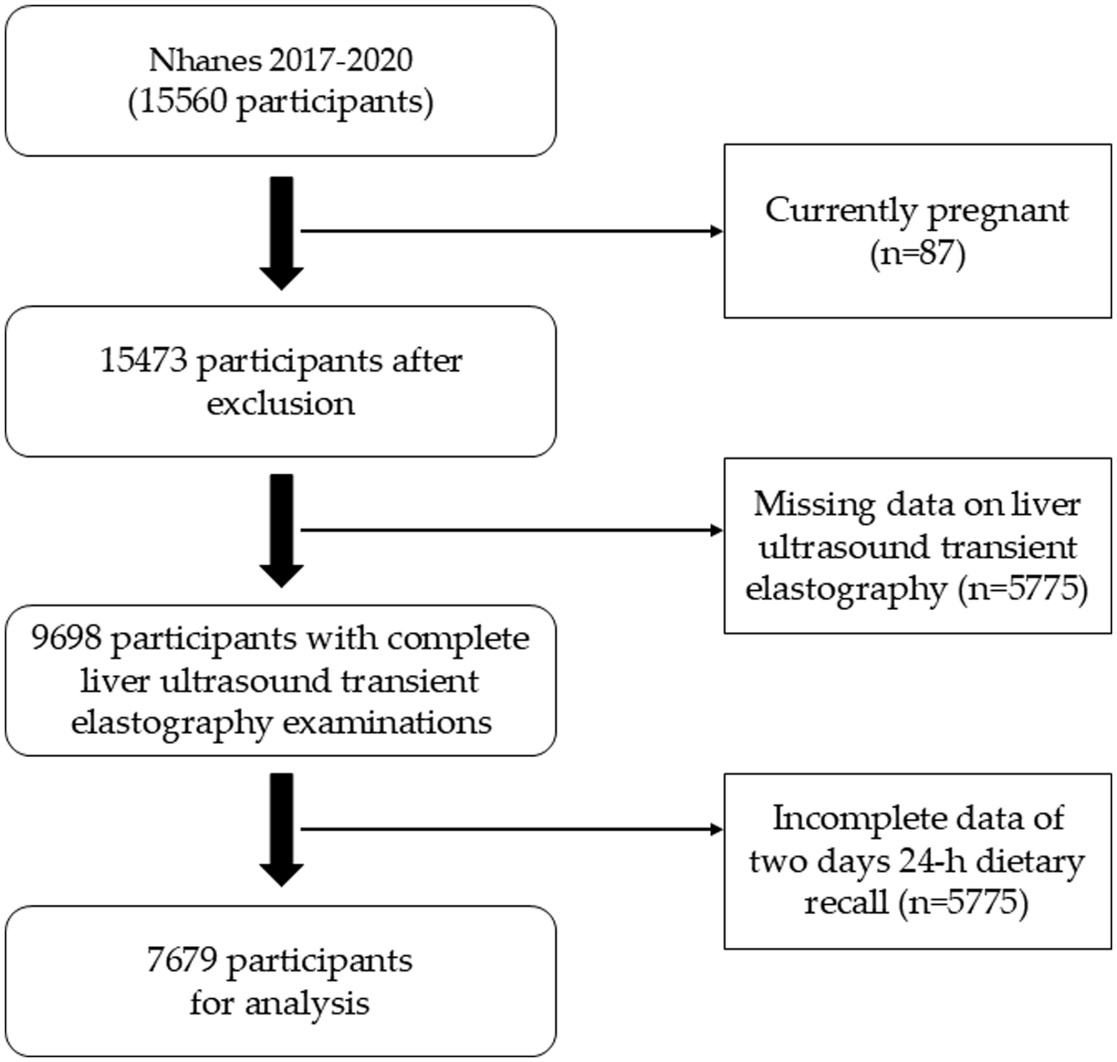
Selection process flowchart for study participants.

The databases were obtained from the NHANES website.^1^ We analyzed the data from the NHANES cycles of 2017–March 2020. A total of 15,560 participants were included. Those currently pregnant (*n* = 87) were excluded from the analysis. 5,862 participants without CAP or LSM measurement were excluded in the study. The participants lack of two-day dietary survey data were also excluded. Finally, our study enrolled 7,679 participants. Detailed study flow was shown as Figure 1.

### 2.2 Study variables

The outcome variables CAP and LSM were evaluated by liver ultrasonographic transient elastography using the FibroScan-equipped model 502 V2 Touch. CAP values ≥274 dB/m was considered indicative of non-steatosis NAFLD status, while CAP ≥302 dB/m was defined as severe steatosis (12, 13). Fibrosis grade was determined by LSM with values of 8.2, 9.7, and 13.6 kPa (13, 20). Serum specimens were taken and stored frozen to obtain biochemical evaluations, then shipped to the University of Minnesota, Minneapolis, MN, United States for further analysis.

The laboratory parameters encompassed plasma triglyceride (TG), total cholesterol (TC), fasting plasma glucose (FPG) and Glycohemoglobin A1c (HbA1c). Demographic data including age, gender, race, education, marital status, poverty income ratio (PIR), alcohol consumption, smoking habits, and medication usage, were collected through standardized questionnaires. Educational levels were categorized into three types: “less than high school,” “high school or equivalent,” and “higher than high school.” Marital status was categorized into three types: “never married,” “married/cohabiting,” or “separated/divorced/widowed.” PIR was classified as three categories: <1.30, 1.30–3.50, and > 3.50 (21). Alcohol consumption was delineated into three groups: individuals who never consumed alcohol, moderate drinkers (1–2 drinks per day for males, 1 drink per day for females), and heavy drinkers (≥3 drinks per day for males, ≥2 drinks per day for females) (21, 22). Smoking habits were categorized into three tiers: low (serum cotinine < 0.015 ng/mL), moderate (0.015–3 ng/mL), and high level (serum cotinine >3 ng/mL) (22, 23).

### 2.3 Dietary assessment

The dietary nutrients consumption was assessed using a 24-h dietary recall method conducted by trained interviewers, who are fluent in Spanish and English. Two dietary assessments were conducted for each participant, with a time interval of 3 to 10 days between them. The first assessment was conducted face-to-face, while the second assessment was conducted via telephone. The setting of the interview is a private room in the Mobile Examination Center (MEC). Each MEC dietary interview room contains a standard set of measuring guides. These tools are used to help the respondent report the volume and dimensions of the food items consumed. They are not intended to represent any one particular food, but rather are designed to help respondents estimate portion sizes. This set of measuring guides is designed specifically for use in the current NHANES setting with a target population of non-institutionalized U.S. civilians. The tools are helpful in portion size estimation for a wide variety of foods (24). We choose nutrients which had established cut points. 34 energy and nutrient intake items were estimated by 24-h dietary recall method. The normal range of each parameter is shown in Supplementary materials. These cut points were taken from a standard textbook, the Dietary Reference Intake (DRIs), published guidelines, and previous studies (25–32).

### 2.4 Statistical analyses

The demographic and abnormal nutrients intakes characteristics are presented as mean ± standard deviation (SD) for continuous variables and as frequency (%) for binary or categorical variables. All values of the data were weighted by weights provided by NHANES. Dietary nutrient consumptions were applied to natural log transformed to address the right-skewed distribution approximate a normal distribution. Descriptive statistics of outcome were presented according to the categories of CAP and LSM. Subgroup differences were explored by chi-square, ANOVA, or Kruskal–Wallis H-test as appropriate. Variables, with different distribution between groups column-stratified by outcomes variables, were further analyzed by multiple linear regression.

Multiple linear regression analysis was used to assess the associations between each dietary nutrient consumption and hepatic steatosis and liver cirrhosis. We formulated three distinct models were to assess the correlation between covariates and the outcomes. Model 1 remained unadjusted, while Model 2 included partial adjustments for age, race, gender, PIR, marital status, and educational level. Model 3 were adjusted for potential covariates including age, gender, race, marriage status, education level, PIR, smoking habits, drinking status, daily energy consumption, TC, TG, Glycohemoglobin and FPG. Energy and dietary fiber per energy intakes were not adjusted for energy intake. The analysis accounted for various covariates and employed restricted cubic spline analysis (RCS) to examine potential nonlinear relationships, when no linear association was found. Spearman’s correlation analysis was conducted to evaluate associations among dietary nutrients. All dietary nutrients were integrated into the weighted quantile sum (WQS) model to examine the collective impact of nutrient mixtures on hepatic steatosis and liver cirrhosis (33). All statistical analyses were performed using R version 4.4.1. A *p*-value below 0.05 was considered as statistically significant.

## 3 Results

### 3.1 Baseline characteristics

15,560 participants from 2017 to March 2020 were included in the study. 5,862 participates were removed participants for lacked of Liver ultrasound transient elastography results. Then, 2019 participates were excluded who did not complete the two-day 24-h dietary survey. Finally, a total of 7,679 subjects met the criteria for inclusion in this analysis (Figure 1). Table 1 showed the baseline characteristics of the participants column-stratified by CAP. Severe hepatic steatosis is more likely to be men, older, married/cohabiting and intake higher energy daily. As to race, Non-Hispanic White and Mexican Americans have a larger proportion. In addition, the non-steatosis group have lower TG, TC, FPG and glycohemoglobin.

**Table 1:** Demographic characteristics of participants by CAP.

Characteristics	Non-steatosis (CAP < 274, *n* = 4,631)	Moderate steatosis(274 ≤ CAP < 302, *n* = 1,083)	Severe steatosis (CAP ≥ 302, *n* = 1965)	*p* value
Age (mean (SD))	40.32 ± 19.68	49 ± 17.99	50.11 ± 17.25	<0.001
Gender (%)				
Male	45.0	51.2	57.7	<0.001
Female	55.0	48.8	42.3	
Race (%)				
Mexican American	7.6	13.6	11.0	<0.001
Other Hispanic	7.8	7.1	8.3	
Non-Hispanic White	61.3	59.0	61.7	
Non-Hispanic Black	13.3	10.4	8.6	
Other Race	10.0	9.9	10.3	
Education (%)				
Less than high school	2.5	2.8	3.1	0.679
High school	6.3	7.3	6.7	
More than high school	91.2	89.9	90.2	
Marriage (%)				
Married/cohabiting	57.6	67.8	69.4	0.02
Widowed/divorced/separated	18.4	19.2	15.6	
Never married	24.0	13.1	15.1	
Poverty income ratio (*n*, %)				
Low level	20.2	18.3	21.0	0.267
Moderate level	33.5	32.9	36.3	
High level	46.2	48.8	42.7	
Cotinine (%)				
Low level	37.7	41.7	39.9	0.264
Moderate level	38.0	37.1	38.7	
High level	24.3	21.2	21.5	
Drinking status (%)				
Non	9.0	6.4	9.8	0.315
Moderate	43.9	46.6	46.6	
Heavy	47.0	47.0	43.6	
Energy daily intake	2019.73 ± 825.83	2076.17 ± 811.14	2145.41 ± 828.88	0.009
TC	4.66 ± 1	4.99 ± 1.11	4.88 ± 1.09	<0.001
TG	1.25 ± 0.78	1.74 ± 1.48	2.09 ± 1.43	<0.001
Glycohemoglobin	5.41 ± 0.6	5.67 ± 0.86	6.08 ± 1.27	<0.001
Glucose	5.08 ± 0.98	5.52 ± 1.64	6.16 ± 2.45	<0.001

CAP, controlled attenuation parameter; SD, standard deviation; Edu, education status; Mari, marital status.

As is shown in Table 2, the cirrhosis group is also more likely to be men and older. The differences in race and daily energy intake level are not as significant as in hepatic steatosis. The analysis of poverty income ratio showed severe liver fibrosis group is less likely to be high income group. The non-steatosis group more likely have lower TG, FPG and glycohemoglobin. The difference in TC is not as significant as for different hepatic steatosis groups.

**Table 2:** Demographic characteristics of participants by LSM.

Characteristics	Non-fibrosis (LSM < 8.2) *n* = 6,922	Significant fibrosis (8.2 < = LSM < 9.7) *n* = 260	Advanced fibrosis (9.7 < = LSM < 13.6) *n* = 264	Cirrhosis (LSM > = 13.6) *n* = 232	*p* value
Age (mean (SD))	43.48 ± 19.40	49.06 ± 19.30	51.72 ± 18.36	50.79 ± 16.92	<0.001
Gender (%)					
Male	47.9	61.1	61.8	64.2	<0.001
Female	52.1	38.9	38.2	35.8	
Race (%)					
Mexican American	9.4	9.5	9.6	7.1	0.781
Other Hispanic	7.9	6.5	7.3	7.5	
Non-Hispanic White	61.1	59.4	61.5	62.5	
Non-Hispanic Black	11.6	14.7	14.4	9.5	
Other Race	10.1	10	7.3	13.4	
Education (%)					
Less than high school	2.6	4.1	6.6	1.9	0.039
High school	6.5	6.3	6.2	10.1	
More than high school	91	89.6	87.2	88	
Marriage (%)					0.585
Married/cohabiting	62.5	61.4	63.2	61.6	
Widowed/divorced/separated	17.3	21.4	22.8	21.4	
Never married	20.2	17.2	14	16.9	
Poverty income ratio (*n*, %)					
Low level	20.2	19.6	21.6	18.5	0.029
Moderate level	33.2	40.7	43.1	47.5	
High level	46.6	39.6	35.3	34	
Cotinine (%)					
Low level	39.4	26.6	33.7	39.4	0.288
Moderate level	37.8	49	39.9	33.8	
High level	22.8	24.4	26.5	26.9	
Drinking status (%)					
Non	8.8	9.7	9.5	6.7	0.831
Moderate	44.9	51.2	42.7	48.3	
Heavy	46.3	39.2	47.8	45	
Energy daily intake	2048.15 ± 817.04	2149.99 ± 861.60	2181.97 ± 940.07	2234.50 ± 918.95	0.02
TC	4.76 ± 1.03	4.65 ± 1.17	4.88 ± 1.26	4.66 ± 1.09	0.599
TG	1.51 ± 1.11	2.05 ± 1.94	2.01 ± 1.44	1.85 ± 1.12	<0.001
Glycohemoglobin	5.57 ± 0.84	6.19 ± 1.37	6.17 ± 1.39	6.19 ± 1.09	<0.001
Glucose	5.34 ± 1.53	6.17 ± 2.32	6.38 ± 2.74	6.39 ± 2.29	<0.001

LSM, liver stiffness measurement; SD, standard deviation; Edu, education status; Mari, marital status.

### 3.2 Associations with CAP

The participants column-stratified by CAP had different distribution of abnormal consumption of carbohydrate, dietary fiber per energy, cholesterol, thiamin, riboflavin, cobalamin, calcium, selenium and caffeine (Table 3). Then Table 4 presented the associations between daily nutrients consumption with CAP by a series of multiple linear regression models. The negative correlations were showed between low carbohydrate, low vitamin C, low vitamin E, pyridoxine, magnesium, iron and potassium intake with CAP only in Model 3 (*p* = 0.011, 0.039, 0.009, 0.024, 0.016, 0.012, and 0.018, respectively). Low dietary fiber per energy intake showed the negative correlation with CAP both in Model 2 and 3 (*p* = 0.006 and 0.005). High percentage of fat and high cholesterol intake showed the positive correlation with CAP both in Model 1 and 2 but not in Model 3 after adjusting all covariates (*p* = 0.001, 0.033, 0.063, <0.001, 0.014, and 0.200, respectively). Low vitamin D, low protein intake, high protein intake was only showed negative correlation with CAP only in Model 1 (*p* = 0.010, 0.017, and 0.043, respectively), the correlation is no longer significant after adjusting covariates. High sodium intake was only showed positive correlation with CAP only in Model 1 (*p* = 0.029). In Model 1, Model 2 and Model3, significant positive correlation was consistently evident between high alcohol intake and low fish oil with CAP (*p* < 0.001, 0.012, 0.030, <0.001, <0.001, and 0.028, respectively).

**Table 3:** Number of participants with abnormal range of daily nutrient intakes by CAP.

Nutrients, N (%)*		Non-steatosis (CAP < 274, *n* = 4,631)	Moderate steatosis (274 ≤ CAP < 302, *n* = 1,083)	Severe steatosis (CAP ≥ 302, *n* = 1965)	*p* value
Energy	Low	28.2	27.8	25.8	0.179
	High	36.0	39.0	41.2	
Protein	Low	16.4	15.0	15.6	0.314
	High	49.5	47.1	52.4	
Carbohydrate	Low	32.4	46.9	51.6	0.008
Simple sugar	High	94.8	94.2	93.6	0.348
Dietary fiber per energy	Low	91.1	94.9	94.3	0.001
Percentage of fat	Low	1.6	1.6	2.0	0.692
	High	60.0	57.8	57.5	
Percentage of saturated fat	High	71.9	71.0	72.6	0.836
Cholesterol	High	37.8	43.5	46.5	0.004
Vitamin A, RAE	Low	75.5	77.6	78.4	0.124
	High	0.1	0.3	0.5	
Vitamin C	Low	64.1	68.5	69.9	0.067
	High	0.0	0.0	0.0	
Vitamin D	Low	72.5	69.2	69.7	0.273
Vitamin E	Low	86.2	91.5	90.6	0.007
Vitamin K	Low	80.5	76.4	76.8	0.259
Thiamin	Low	23.5	27.6	28.5	0.009
Riboflavin	Low	33.0	24.6	25.5	0.005
Niacin	Low	18.8	15.8	17.4	0.806
	High	11.8	11.1	14.0	
Pyridoxine	Low	35.1	33.1	35.0	0.815
Folate	Low	46.7	44.8	45.7	0.145
	High	5.1	3.2	3.9	
Cobalamin	Low	28.2	24.5	21.8	0.014
Calcium	Low	29.9	36.0	37.8	0.002
	High	0.8	1.3	0.6	
Phosphorous	Low	9.6	7.3	7.9	0.204
	High	0.0	0.0	0.0	
Magnesium	Low	77.5	81.1	79.3	0.302
Iron	Low	27.4	31.1	27.8	0.241
	High	0.7	0.2	0.5	
Zinc	Low	49.6	49.4	45.8	0.179
	High	0.1	0.1	0.2	
Copper	Low	39.4	35.4	35.6	0.103
	High	0.0	0.1	0.3	
Sodium	Low	4.5	4.2	4.3	0.007
	High	74.3	76.7	80.6	
Potassium	Low	96.4	97.2	96.9	0.601
Selenium	Low	10.5	9.4	7.3	0.021
	High	0.1	0.0	0.1	
Caffeine	High	5.1	9.9	9.0	0.001
Alcohol	High	13.1	17.9	15.2	0.213
Linoleic acid	Low	38.6	40.5	35.2	0.222
α-Linolenic acid	Low	37.7	38.6	37.0	0.887
Fish oil	Low	93.0	90.8	93.3	0.310

CAP, controlled attenuation parameter.

**Table 4:** Linear regression model between daily nutrients consumption and CAP.

Nutrients		Model 1		Model 2		Model 3	
		β-coefficient (95%CI)	p trend	β-coefficient (95%CI)	p trend	β-coefficient (95%CI)	p trend
Energy	Low	4.1(−11, 19)	0.600	−6.1(−29, 17)	0.600	−2.9(−25, 19)	0.800
	High	20(−2.8, 42)	0.083	−0.71(−27, 26)	>0.900	−1.5(−30, 27)	>0.900
Protein	Low	18(3.5, 32)	0.017	16(0.04, 32)	0.050	3.6(−16, 23)	0.700
	High	16(54, 32)	0.043	6.2(−12, 25)	0.500	−2.9(−23, 17)	0.800
Carbohydrate	Low	−3.1(−15, 8.5)	0.600	−6.5(−20, 6.8)	0.300	−14(−25, −4.0)	0.011
Simple sugar	High	2.6(−0.51, 5.6)	0.100	1.3(−1.9, 4.5)	0.400	−0.82(−7.6, 5.9)	0.800
Dietary fiber per energy*	Low	−8.9(−18, 0.02)	0.050	−17(−28, −5.4)	0.006	−21(−34, −7.7)	0.005
Percentage of fat	Low	−19(−68, 31)	0.400	−8.7(−61, 44)	0.700	−2.2(−74, 70)	>0.900
	High	39(17, 61)	0.001	25(2.3, 48)	0.033	27(−1.7, 55)	0.063
Percentage of saturated fat	High	13(−5.5, 32)	0.200	7.0(−10, 24)	0.400	12(−8.5, 32)	0.200
Cholesterol	High	8.6(5.2, 12)	<0.001	5.3(1.2, 9.4)	0.014	2.7(−1.8, 7.1)	0.200
Vitamin A, RAE*	Low	3.9(−1.2, 8.9)	0.130	0.20(−5.8, 6.2)	>0.900	−3.1(−8.3, 2.2)	0.200
Vitamin C*	Low	0.26(−3.3, 3.9)	0.900	−3.5(−7.5, 0.48)	0.081	−4.8(−9.4,-0.30)	0.039
Vitamin D*	Low	10(2.6, 17)	0.010	4.9(−3.9, 14)	0.300	0.13(−10, 11)	>0.900
Vitamin E	Low	1.1(−5.7, 7.9)	0.700	−3.9(−11, 3.0)	0.300	−11(−19, −3.5)	0.009
Vitamin K	Low	4.9(−0.23, 10)	0.060	0.82(−4.3, 5.9)	0.700	1.5(−4.6, 7.6)	0.600
Thiamin	Low	5.7(−5.6, 17)	0.300	−2.0(−17, 13)	0.800	−9.9(−28, 8.6)	0.300
Riboflavin	Low	9.8(−6.1, 26)	0.200	−4.2(−19, 11)	0.600	−19(−39, 2.0)	0.072
Niacin	Low	8.9(−7.5, 25)	0.300	−1.9(−24, 20)	0.900	−18(−58, 22)	0.300
	High	−5.0(−32, 22)	0.700	−0.45(−30, 29)	>0.900	−0.72(−29, 31)	>0.900
Pyridoxine	Low	11(1.5, 20)	0.025	−2.0(−15, 11)	0.800	−19(−36, −2.8)	0.026
Folate	Low	2.1(−6.8,11)	0.600	−2.0(−13, 9.4)	0.700	−9.3(−23, 4.7)	0.200
Cobalamin	Low	10(−6.3, 27)	0.200	10(−11, 32)	0.300	10(−19, 40)	0.500
Calcium	Low	−2.9(−14, 8.5)	0.600	−7.3(−20, 5.8)	0.300	−17(−34, 0.07)	0.051
Phosphorous	Low	−1.9(−24, 21)	0.900	−2.8(−23, 17)	0.800	1.0(−35, 37)	>0.900
Magnesium	Low	11(3.7, 19)	0.005	−3.7(−13, 5.9)	0.400	−16(−29, −3.6)	0.016
Iron	Low	−7.5(−20, 5.1)	0.200	−3.5(−20, 13)	0.700	−27(−47, −7.4)	0.012
Zinc	Low	15(5.1, 25)	0.005	3.0(−9.5, 15)	0.600	−8.7(−22, 5.0)	0.200
Copper	Low	4.5(−13, 22)	0.600	1.7(−19, 23)	0.900	−11(−30, 8.4)	0.200
Sodium	Low	4.0(−35, 43)	0.800	14(−24, 52)	0.500	11(−37, 59)	0.600
	High	12(1.3, 22)	0.029	10(−1.9, 22)	0.094	8.3(−7.9, 24)	0.300
Potassium	Low	12(5.0, 18)	0.001	−2.4(−10, 5.5)	0.500	−16(−28, −3.3)	0.018
Selenium	Low	4.5(−15, 24)	0.600	3.2(−18, 24)	0.700	−10(−29, 8.6)	0.300
Caffeine	High	26(−14, 66)	0.200	18(−23, 58)	0.400	15(−35, 64)	0.500
Alcohol	High	28(17, 38)	<0.001	19(4.7, 33)	0.012	17(1.9, 31)	0.030
Linoleic acid	High	−0.52(−8.5, 7.4)	0.900	0.35(−11, 12)	>0.900	−4.4(−20, 12)	0.600
α-Linolenic acid	Low	6.9(−1.8, 16)	0.110	2.3(−9.0, 14)	0.700	1.6(−11, 14)	0.800
Fish oil*	Low	111(68, 153)	<0.001	96(50, 142)	<0.001	87(11, 163)	0.028

Dietary nutrient intakes were log-transformed. CAP, controlled attenuation parameter. *Log(x+1) transformed for variables containing zeros.

### 3.3 Associations with LSM

Table 5 revealed the outcomes derived from multiple logistic regression models scrutinizing the potential independent associations between 34 abnormal nutrients consumption and LSM. Protein, vitamin A, cholesterol, calcium and Copper’ abnormal consumption showed different distribution among participants groups column-stratified by LSM (Table 5). Table 6 presented the associations between protein per weight and cobalamin with LSM by a series of multiple linear regression models. The negative correlations were showed between low protein intake, low dietary fiber per energy, low vitamin A, low vitamin E, pyridoxine, phosphorous, magnesium, zinc, copper and potassium intake with LSM in Model 3 (*p* = 0.001, 0.009, 0.016, 0.012, <0.001, 0.048, 0.003, 0.002, 0.002, and 0.012, respectively). High caffeine intake showed the positive correlation with LSM in Model 3 after adjusting all covariates (*p* = 0.022).

**Table 5:** Number of participants with abnormal range of daily nutrient.

Nutrients, N (%)*		Non-fibrosis (LSM < 8.2) *n* = 6,922	Significant fibrosis (8.2 < = LSM < 9.7) *n* = 260	Advanced fibrosis (9.7 < = LSM < 13.6) *n* = 264	Cirrhosis (LSM > = 13.6) *n* = 232	*p* value
Energy	Low	27.6	24.8	30.3	25.5	0.498
	High	37.3	41.8	44.4	41.6	
Protein	Low	16	14.9	15	16.2	0.027
	High	49.3	52.2	51.3	65	
Carbohydrate	Low	30.7	27.6	29.2	28.4	0.801
Simple sugar	High	94.6	94.5	89.9	92.2	0.048
Dietary fiber per energy	Low	92.4	97.5	94.6	89.7	0.330
Percentage of fat	Low	1.6	2.1	2.2	4.7	0.058
	High	58.5	59.6	68.2	64.4	
Percentage of saturated fat	High	71.7	71.3	81.3	71	0.224
Cholesterol	High	40.1	42.7	49.6	54.8	0.011
Vitamin A, RAE	Low	76.7	75.0	80.2	70.4	0.011
	High	0.2	0.4	0	2.9	
Vitamin C	Low	66.3	61.2	64.1	71.7	0.441
	High	0	0	0	0	
Vitamin D	Low	71.4	70.7	69.1	69.9	0.931
Vitamin E	Low	88.2	82.6	90.3	87.6	0.517
Vitamin K	Low	78.7	80.4	75.9	74.6	0.571
Thiamin	Low	31.6	25.9	34.9	25.7	0.190
Riboflavin	Low	29.9	25	27.5	25.7	0.433
Niacin	Low	17.4	18.5	20.1	18.2	0.443
	High	11.8	14.8	18.4	17.8	
Pyridoxine	Low	34.7	35.4	40.3	31.4	0.583
Folate	Low	46.3	46	48.3	41.4	0.545
	High	4.7	2.4	4.1	3	
Cobalamin	Low	26.4	21.2	23.7	20.3	0.234
Calcium	Low	32	38.6	38.3	46.7	0.018
	High	0.8	0.7	2.9	1.1	
Phosphorous	Low	8.7	11.2	10.7	7.5	0.443
	High	0	0	0	0	
Magnesium	Low	78.5	82.5	80	74.4	0.754
Iron	Low	27.8	32.3	32.3	26.4	0.714
	High	0.6	0	0.1	0.8	
Zinc	Low	48.6	48.4	50.3	47.3	0.981
	High	0.1	0	0	0	
Copper	Low	38.2	39.5	36.7	27.3	<0.001
	High	0	0.1	0	2.6	
Sodium	Low	4.5	2.1	3.2	3.4	0.390
	High	76	78	77.4	82.5	
Potassium	Low	96.6	99.3	96.5	94.9	0.255
Selenium	Low	9.5	9.9	8	9.3	0.148
	High	0.1	0	0	0.7	
Caffeine	High	6.9	5	8.1	4.9	0.801
Alcohol	High	14.5	12.3	10.1	13.2	0.624
Linoleic acid	Low	37.9	39.4	42.6	36.4	0.584
α-Linolenic acid	Low	37.5	42.4	40.6	35.5	0.641
Fish oil	Low	92.8	92.9	91.2	91.7	0.771

LSM, liver stiffness measurement.

**Table 6:** Linear regression model between daily nutrients consumption and LSM.

Nutrients		Model 1		Model 2		Model 3	
		β-coefficient (95%CI)	p trend	β-coefficient (95%CI)	p trend	β-coefficient (95%CI)	p trend
Energy	Low	−0.08(−0.78, 0.63)	0.800	−0.67(−2.0, 0.69)	0.300	−0.96(−3.3, 1.4)	0.400
	High	2.0(0.75, 3.3)	0.003	2.2(0.02, 4.3)	0.048	2.3(−0.01, 4.5)	0.051
Protein	Low	−0.59(−1.3, 0.11)	0.095	−1.2(−3.3, 0.95)	0.300	−3.3(−4.9, −1.6)	0.001
	High	1.5(0.80, 2.3)	<0.001	1.5(0.12, 2.9)	0.035	1.1(−1.1, 3.3)	0.300
Carbohydrate	Low	0.21(−0.40, 0.83)	0.500	0.03(−0.72, 0.78)	>0.900	0.41(−1.5, 2.4)	0.700
Simple sugar	High	0.33(0.11, 0.54)	0.005	0.30(0.03, 0.57)	0.032	0.19(−0.20, 0.58)	0.300
Dietary fiber per energy*	Low	−0.79(−1.2, −0.36)	<0.001	−0.84(−1.4, −0.29)	0.005	−1.0(−1.7,-0.30)	0.009
Percentage of fat	Low	1.5(−9.5, 13)	0.800	2.3(−7.6, 12)	0.600	−0.50(−15, 14)	0.900
	High	0.54(−1.5, 2.6)	0.600	0.10(−1.8, 2.0)	>0.900	−0.05(−1.9, 1.8)	>0.900
Percentage of saturated fat	High	0.93(−0.13, 2.0)	0.084	0.88(−0.42, 2.2)	0.200	0.95(−0.53, 2.4)	0.200
Cholesterol*	High	0.31(0.13, 0.48)	0.001	0.29(0.06, 0.51)	0.017	−0.03(−0.28, 0.21)	0.800
Vitamin A, RAE*	Low	−0.18(−0.49, 0.13)	0.200	−0.26(−0.56, 0.04)	0.083	−0.58(−1.0, −0.13)	0.016
Vitamin C*	Low	−0.11(−0.36, 0.14)	0.400	−0.17(−0.42, 0.08)	0.200	−0.28(−0.61, 0.05)	0.089
Vitamin D*	Low	0.14(−0.29, 0.57)	0.500	0.19(−0.29, 0.66)	0.400	0.08(−0.53, 0.68)	0.800
Vitamin E	Low	−0.03(−0.40, 0.35)	0.900	−0.13(−0.67, 0.42)	0.600	−0.65(−1.1, −0.17)	0.012
Vitamin K	Low	0.21(−0.17, 0.58)	0.300	0.06(−0.47, 0.58)	0.800	−0.31(−0.95, 0.34)	0.300
Thiamin	Low	−0.09(−0.77, 0.59)	0.800	−0.27(−1.3, 0.80)	0.600	−0.26(−1.2, 0.66)	0.500
Riboflavin	Low	−0.61(−1.9, 0.73)	0.400	−1.2(−2.2, −0.26)	0.016	−2.1(−4.6, 0.33)	0.083
Niacin	Low	0.61(−0.74, 2.0)	0.400	0.44(−0.76, 1.6)	0.400	−0.16(−3.1, 2.8)	>0.900
	High	0.01(−2.1, 2.1)	>0.900	0.14(−2.4, 2.7)	>0.900	−0.96(−3.3, 1.4)	0.400
Pyridoxine	Low	−0.42(−0.90,0.05)	0.079	−0.69(−1.4,0.06)	0.068	−1.7(−2.5,-0.86)	<0.001
Folate	Low	−0.24(−0.66, 0.18)	0.300	−0.32(−0.90, 0.26)	0.300	−0.42(−1.0, 0.20)	0.200
Cobalamin	Low	−0.42(−1.9,1.0)	0.600	−0.15(−1.5,1.2)	0.800	−0.33(−3.1,2.5)	0.800
Calcium	Low	−0.30(−0.90, 0.29)	0.300	−0.17(−0.82, 0.47)	0.600	−0.08(−1.5, 1.4)	>0.900
Phosphorous	Low	−0.64(−2.2, 0.96)	0.400	−0.43(−1.7, 0.85)	0.540	−2.3(−4.6, −0.02)	0.048
Magnesium	Low	−0.05(−0.58, 0.49)	0.900	−0.47(−1.4, 0.48)	0.300	−1.4(−2.3, −0.59)	0.003
Iron	Low	-0.59(−1.4, 0.18)	0.120	−0.92(−2.5, 0.66)	0.200	−1.8(−3.8, 0.12)	0.063
Zinc	Low	−0.13(−0.80, 0.55)	0.700	−0.71(−1.9, 0.44)	0.200	−2.0(−3.1, −0.94)	0.002
Copper	Low	−1.6(−2.1, −1.1)	<0.001	−2.1(−2.7, −1.5)	<0.001	−2.7(−3.9, −1.4)	<0.001
Sodium	Low	−0.40(−1.6, 0.77)	0.500	0.48(−0.83, 1.8)	0.400	0.23(−1.8, 2.2)	0.800
	High	1.2(0.22, 2.3)	0.019	1.2(−0.27, 2.6)	0.110	0.30(−1.2, 1.8)	0.700
Potassium	Low	0.12(−0.28, 0.53)	0.500	−0.18(−0.81, 0.45)	0.500	−1.3(−2.3, −0.35)	0.012
Selenium	Low	0.37(−0.50, 1.2)	0.400	0.76(−0.37, 1.9)	0.200	0.74(−1.2, 2.7)	0.400
Caffeine	High	0.82(−0.84, 2.5)	0.300	0.97(−0.79, 2.7)	0.300	1.7(0.34, 3.0)	0.022
Alcohol	High	0.77(−0.06, 1.6)	0.068	−0.15(−1.7, 1.4)	0.800	−0.22(−0.52, 0.09)	0.120
Linoleic acid	High	−0.33(−0.92, 0.25)	0.200	−0.69(−1.9, 0.51)	0.200	−0.75(−2.1, 0.56)	0.200
α-Linolenic acid	Low	−0.07(−0.68, 0.53)	0.800	−0.62(−1.6, 0.37)	0.200	−0.78(−1.9, 0.30)	0.140
Fish oil*	Low	1.1(−2.6, 4.7)	0.600	1.3(−3.6, 6.2)	0.600	−2.4(−7.0, 2.2)	0.300

Dietary nutrient intakes were log-transformed. LSM, liver stiffness measurement. *Log(x+1) transformed for variables containing zeros.

### 3.4 Nonlinear relationships

As is shown in Supplementary Figure 1, nonlinear relationships were found between energy, vitamin E, folate, sodium, alcohol, *α*-Linolenic acid and fish oil with hepatic steatosis in the fully adjusted model (*p* < 0.05). The associations of these nutrients with risk of hepatic steatosis changed at these points (energy: 1949 kcal, vitamin E: 7.59 mg, folate: 410mcg, sodium: 3187.5 mg, alcohol: 38.9 g, linoleic acid: 19.7 mg, *α*-linolenic acid: 2.58 g, fish oil: 1.68 g) (Table 7). The intake of most dietary nutrients, including energy, protein, carbohydrate, simple sugars, dietary fiber per energy, percentage of fat, percentage of saturated fat, cholesterol, vitamin A, vitamin E, vitamin K, pyridoxine, cobalamin, magnesium, zinc, copper, potassium, potassium, alcohol and *α*-Linolenic acid, were found to have nonlinear relationships with liver fibrosis (*p* < 0.05) (Supplementary Figure 1). The associations of these nutrients with increasing risk of liver cirrhosis changed at these points (energy: 1976 kcal, protein: 47 g, carbohydrate: 141 g, simple sugars: 16.2 g, percentage of fat: 27%; percentage of saturated fat: 11%; cholesterol: 276 mg; vitamin A: 723mcg, vitamin E: 7.58 mg, vitamin K: 85mcg, riboflavin: 1.06 mg, pyridoxine: 1.14 mg, cobalamin: 3.43 mg, magnesium: 403 mg, zinc: 6.84 mg, copper: 1.02 mg, potassium: 2356 mg, alcohol: 52 g, α-linolenic acid: 2.57 g) (Table 8).

**Table 7:** Nonlinear relationships of the association of hepatic steatosis and nutrients.

Nutrients	Total p trend	Non-linear p trend	Critical value
Energy	<0.0001	0.0214	1949 kcal
Vitamin E	<0.0001	0.0001	7.59 mg
Folate	<0.0001	0.0369	410mcg
Sodium	0.0069	0.0154	3187.5 mg
Alcohol	<0.0001	<0.0001	38.9 g
Linoleic acid	<0.0001	0.0006	19.7 mg
α-Linolenic acid	<0.0001	<0.0001	2.58 g
Fish oil	<0.0001	<0.0001	1.68 g

**Table 8:** Nonlinear relationships of the association of liver fibrosis and nutrients.

Nutrients	Total p trend	Non-linear p trend	Critical value
Energy	0.0049	0.0349	1976 kcal
Protein	0.0100	0.0040	47 g
Carbohydrate	0.0044	0.0062	141 g
Simple sugar	0.0225	0.0087	16.2 g
Percentage of fat	<0.0001	<0.0001	27%
Percentage of saturated fat	<0.0001	<0.0001	11%
Cholesterol	0.0110	0.0187	276 mg
Vitamin A	0.0001	0.0001	723mcg
Vitamin E	<0.0001	<0.0001	7.58 mg
Vitamin K	0.0002	0.0056	85mcg
Riboflavin	0.0043	0.0034	1.06 mg
Pyridoxine	0.0173	0.0087	1.14 mg
Cobalamin	0.0006	0.0004	3.43 mg
Magnesium	<0.0001	<0.0001	403 mg
Zinc	0.0154	0.0065	6.84 mg
Copper	<0.0001	<0.0001	1.02 mg
Potassium	0.0001	0.0395	2,356 mg
Alcohol	0.0041	0.0020	52 g
α-Linolenic acid	0.0068	0.0023	2.57 g

### 3.5 Spearman’s and WQS regression analysis

**Figure 2:**
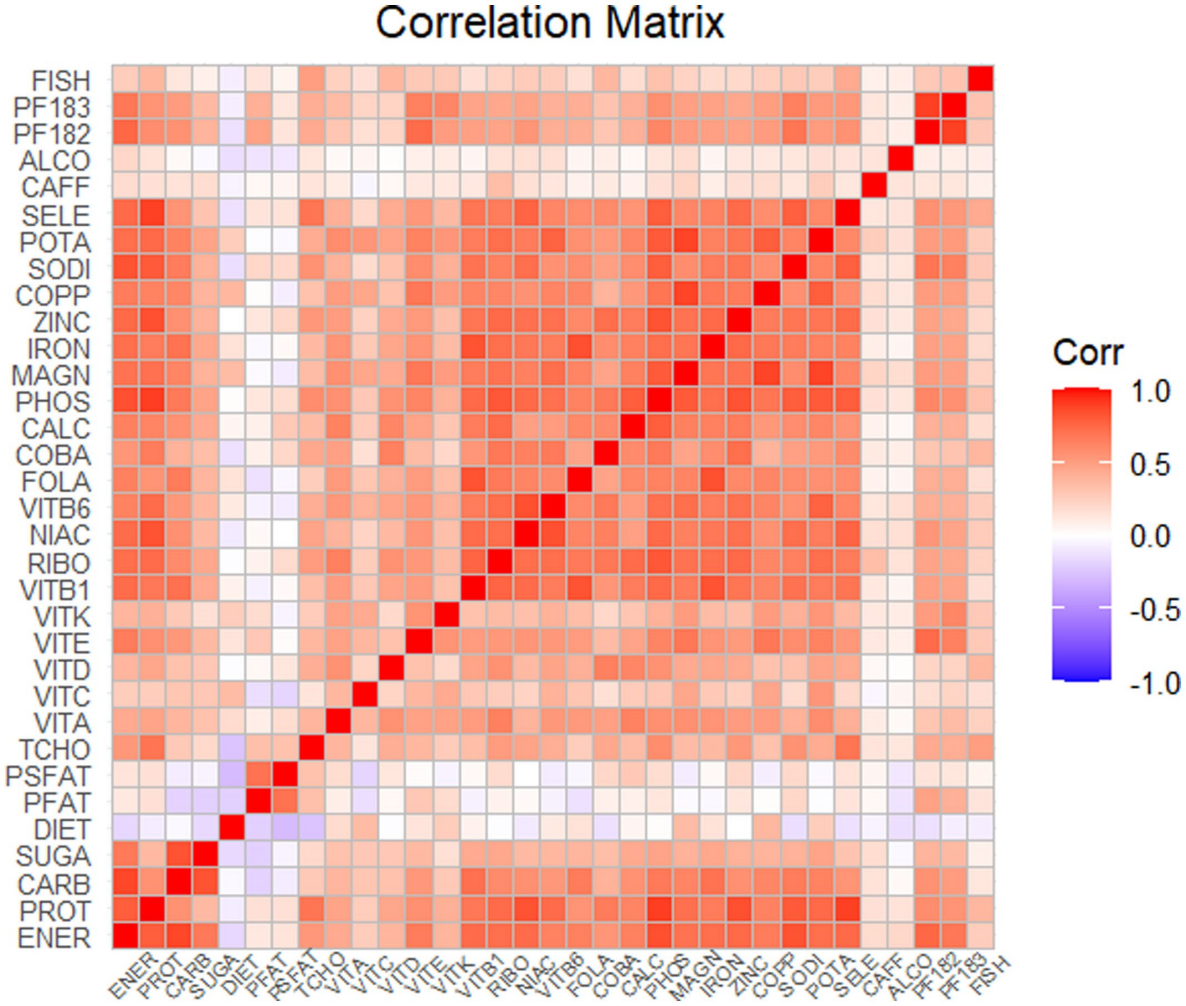
Spearman’s correlation matrix among Log-transformed dietary nutrient.

**Figure 3:**
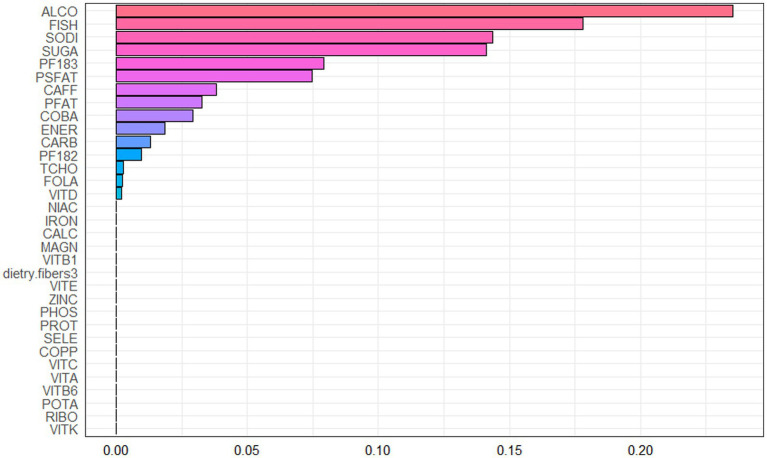
Weighted values of dietary nutrient intakes for CAP in WQS models.

**Figure 4:**
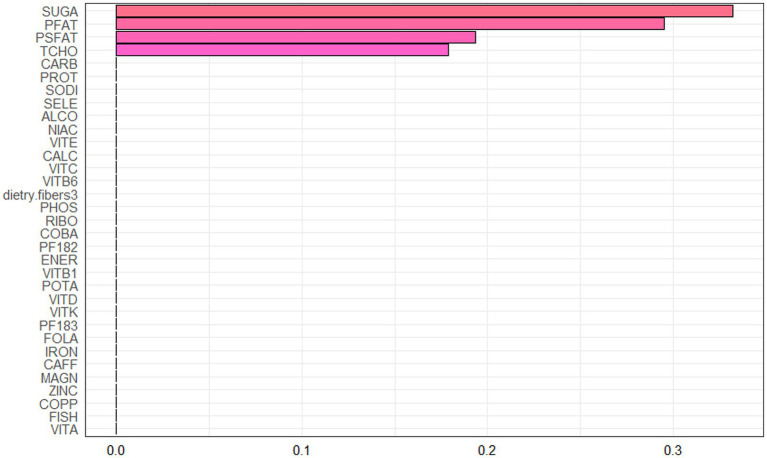
Weighted values of dietary nutrient intakes for LSM in WQS models.

Spearman correlation analysis was used to analyze the association among the dietary nutrients. It revealed that significant positive associations among the majority of dietary nutrients (Figure 2, Supplementary Table 2). The WQS index of mixed dietary nutrients log-transformed intakes was positively associated with hepatic steatosis and liver fibrosis (*β*: 2.312, 95% CI: 0.897–2.578, *p* = 9.985e^−3; β: 0.143, 95% CI: 0.063–2.266, *p* = 0.024; respectively). The top four nutrients with higher weights associated with CAP in the WQS were: alcohol, fish oil, sodium and simple sugar. The top four nutrients with higher weights associated with LSM in the WQS were: simple sugar, percentage of fat, percentage of saturated fat and total cholesterol (Figures 3, 4).

## 4 Discussion

With the development of economy, the incidence of chronic liver disease has gradually increased and become a global health concern (34–37). The liver plays a major role in nutrient metabolism, including digestion, absorption, storage, synthesis and a series of physiological processes (38). The dysfunction of liver disrupt the homeostasis of nutrition metabolic homeostasis. Liver diseases include NAFLD, cirrhosis and liver failure could not only affect the metabolism of glucose and lipid, resulting in fat accumulation and protein deficiency, but also affect the metabolism a series of vitamins and microelements (39, 40).

The association between nutrition and liver diseases has been well recognized. This correlation exhibits bidirectional characteristics. On the one hand, recent studies have revealed that nutrition has a central prognostic and therapeutic role in the management of patients with liver disease (41). On the other hand, malnutrition and nutritional metabolic disorders is common in patients with liver disease and has a significant impact on disease progression and prognosis (42–44). Nutrition has been recognized as independent predictors of a higher rate of complications and lower survival in liver diseases (45–48).

Dietary nutrition is an important aspect of alimentology. Dietary patterns was revealed to have significant impacts on hepatic steatosis (17). As to liver cirrhosis, inadequate dietary intake seemed to be a self-dependent predictor of in-hospital mortality and affect progressive liver failure (49). The roles of various nutrients in this context have drawn attention. Current limited research findings indicate that the relationship between macronutrients and their effects remains controversial, particularly evident in the case of protein liver cirrhosis (14–17, 50, 51). As for micronutrients, the liver is important for their metabolism but the role of micronutrients in NAFLD remains less known (18, 33). Several micronutrients, such as zinc, copper, iron, selenium, magnesium, vitamins A, C, D, and E seems to have beneficial effects in NAFLD, however, the conclusions remain controversial and the appropriate dosage appears to be important but unclear (52, 53). Excess intake of some micronutrients such as iron and selenium may increase the severity of NAFLD as reported (54–56). Dissecting the specific contributions of micronutrients, however, remains challenging because human diets are complex and fail to replicate experimental dietary models (33). Exploring appropriate intake levels remains a critical research gap in current scientific investigations. In terms of liver fibrosis, zinc, vitamin D, vitamin E, copper and iron garnered research attention (57–61). Nevertheless, there is still a lack of comprehensive systematic research on the role of daily dietary nutrients in hepatic steatosis and liver fibrosis.

Thus, we conducted the study by analyzing the latest NHANES cycle. We used liver ultrasonographic transient elastography to monitor noninvasive hepatic steatosis and liver fibrosis. Daily dietary nutrient intake data were obtained through a standardized 24-h dietary survey to explore the associations between comprehensive dietary nutrients consumption and hepatic steatosis and liver fibrosis. Our study had discovered a correlation between some previously overlooked nutrients and NAFLD, such as pyridoxine, magnesium, potassium intake, linoleic acid,*α*-Linolenic acid. Nonlinear correlation was found to exist between the consumption of energy, vitamin E, folate, sodium, alcohol, α-Linolenic acid and fish oil and hepatic steatosis. High caffeine intake surprisingly showed the positive correlation with LSM in Model 3 after adjusting all covariates. We identified that intakes of many nutrients had significant nonlinear associations with hepatic fibrosis. The correlation between dietary nutrients and disease showed actually more complex relationship. RCS curves helped to reveal the recommended nutritional intake threshold to prevent hepatic steatosis. Energy less than 1,949 kcal, vitamin E higher than 7.59 mg, folate higher than 410mcg, sodium lower than 3187.5 mg, alcohol lower than 38.9 g, fish oil higher than 1.68 g 1 day, etc. may be the recommended consumption for fatty liver. It can be seen that these doses are not exactly the same as our daily RNI or UI values, which may provide the evidence to build a precise diseases-oriented nutrition strategy. The consumption such as fish oil’s benefits can only obtain when reaching a certain intake level, which exceed the daily intake level. Additional supplements may be necessary based on the evidence. As to liver fibrosis, enough nutrients intake was important, for example, protein intake higher than 47 g/d and carbohydrate higher than 141 g. In addition, micronutrients such as vitamin A, vitamin E, magnesium, zinc, copper also played their roles. RCS curves also revealed the recommended nutritional intake threshold as mentioned in the article. In contrast to previous studies, our research has identified that the majority of nutrient intake still follows a threshold effect. For certain nutrients previously considered of critical importance, such as vitamin D, alterations in daily intake do not appear to effectively reduce disease risks. These findings have provided us with significant insights.

Several limitations should be acknowledged in our study. This article involved a wide range of dietary nutrients providing guidance on dietary nutrients for hepatic steatosis and liver cirrhosis. However, it is limited by its excessive coverage, then many questions need further explore refined to each specific nutrient. Moreover, nutrition and liver damage, who is the cause? Who is the outcome? A positive or negative correlation is not enough. The status of severe steatosis and fibrosis may affect the dietary habit through affecting digestive function and feeding center. This is another issue worth exploring and considering. Large-scale prospective cohort studies, statistical analysis such as Mendelian analysis or more complex mathematical modeling methods are needed to help us further explore the relationship between nutrients and hepatic steatosis and liver fibrosis. By precise dietary adjustment, we may obtain more health benefits on our liver health.

## 5 Conclusion

The study identified various dietary nutrients associated with hepatic steatosis and liver fibrosis. Nonlinear correlations were observed between the consumption of energy, vitamin E, folate, sodium, alcohol, *α*-linolenic acid, fish oil, and hepatic steatosis. Similarly, liver fibrosis showed nonlinear relationships with the intake of multiple nutrients, including energy, protein, carbohydrates, simple sugars, dietary fiber per energy, fat percentage, saturated fat percentage, cholesterol, vitamins (A, E, K, pyridoxine, cobalamin), and minerals (magnesium, zinc, copper, potassium). Critical threshold intake levels in associated nutrients were revealed that may elevate disease risk. The impact of abnormal nutrient consumption appeared to be more pronounced in hepatic steatosis. These findings may help us better understand the complex relationship between diet and hepatic steatosis and fibrosis. Moreover, this data provides critical insights for establishing evidence-based clinical nutrition strategies to optimize the prevention and management of liver diseases. While recommended nutrient intake for liver conditions was identified, further research is needed to explore the effects of nutritional interventions and clarify causal relationships in chronic liver diseases.

## Data Availability

Publicly available datasets were analyzed in this study. This data can be found here: the databases were obtained from the NHANES website (https://www.cdc.gov/nchs/nhanes/index.htm).

## Footnotes

^1^ https://www.cdc.gov/nchs/nhanes/index.htm
