# Supplementary material for: The association of dietary nutrients consumption with hepatic steatosis and fibrosis from NHANES 2017–2020
Source: Front Nutr. 2025 Jun 24;12:1510860. doi: 10.3389/fnut.2025.1510860 (PMC12234298; doi:10.3389/fnut.2025.1510860)
Supplement: Supplementary file 1 [file Image_1.pdf]

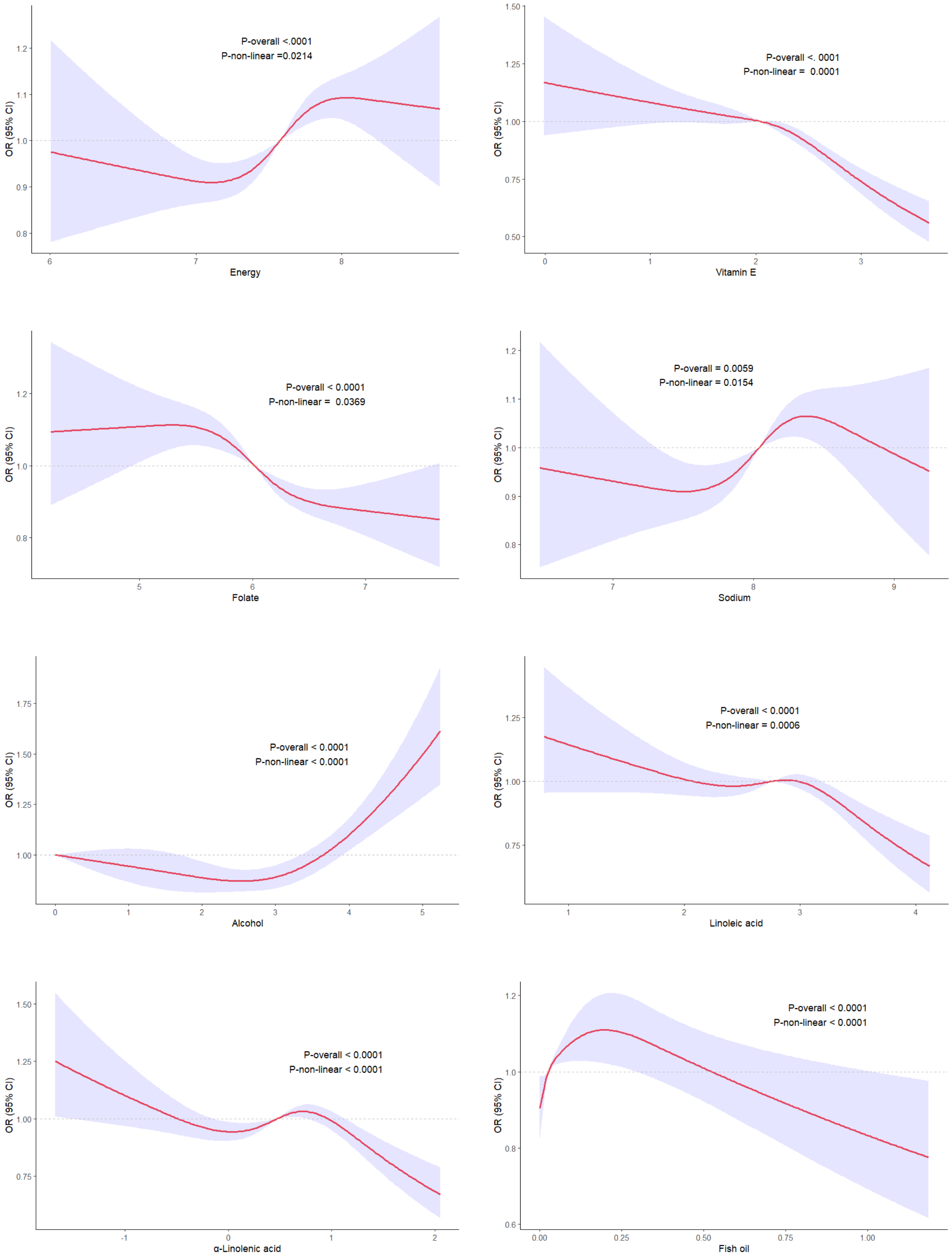

Figure S1. Restricted cubic spline plots of the association of hepatic steatosis and nutrients.

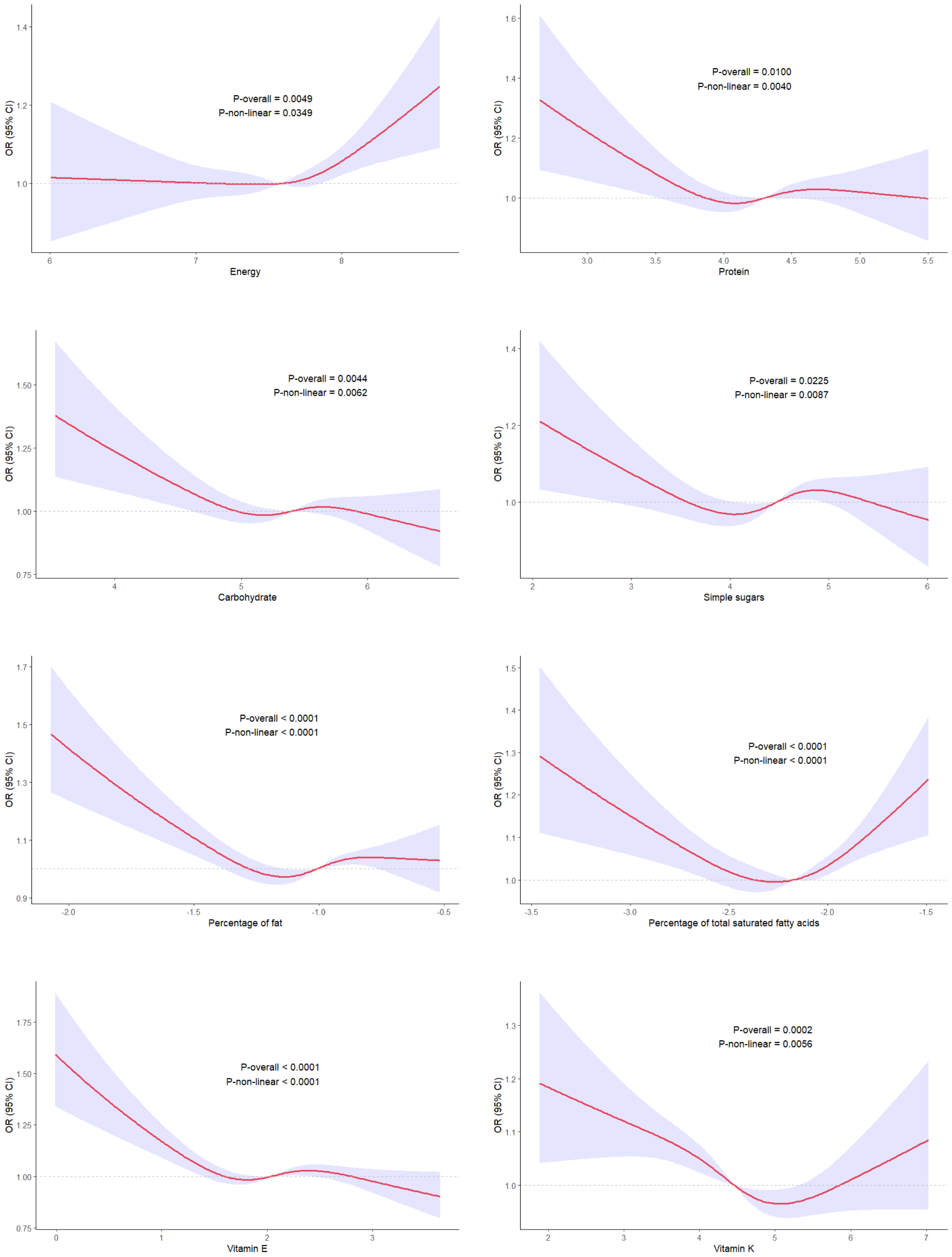

OR (95% CI)
P-overall = 0.0049
P-non-linear = 0.0349
Energy
P-overall = 0.0100
P-non-linear = 0.0040
Protein
P-overall = 0.0044
P-non-linear = 0.0062
Carbohydrate
P-overall = 0.0225
P-non-linear = 0.0087
Simple sugars
P-overall < 0.0001
P-non-linear < 0.0001
Percentage of fat
P-overall < 0.0001
P-non-linear < 0.0001
Percentage of total saturated fatty acids
P-overall < 0.0001
P-non-linear < 0.0001
Vitamin E
P-overall = 0.0002
P-non-linear = 0.0056
Vitamin K

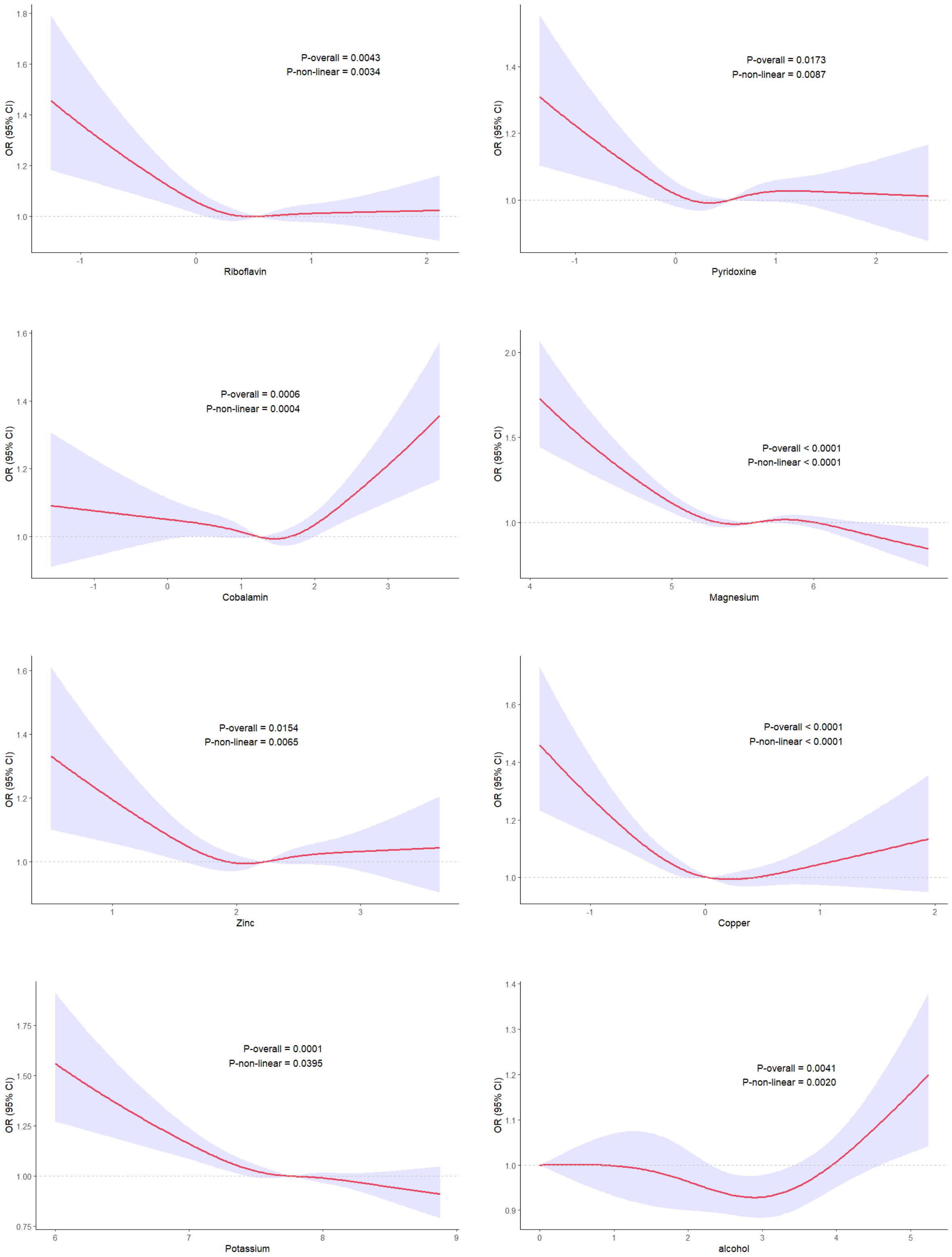

P-overall = 0.0043
P-non-linear = 0.0034
OR (95% CI)
Riboflavin
P-overall = 0.0173
P-non-linear = 0.0087
OR (95% CI)
Pyridoxine
P-overall = 0.0006
P-non-linear = 0.0004
OR (95% CI)
Cobalamin
P-overall < 0.0001
P-non-linear < 0.0001
OR (95% CI)
Magnesium
P-overall = 0.0154
P-non-linear = 0.0065
OR (95% CI)
Zinc
P-overall < 0.0001
P-non-linear < 0.0001
OR (95% CI)
Copper
P-overall = 0.0001
P-non-linear = 0.0395
OR (95% CI)
Potassium
P-overall = 0.0041
P-non-linear = 0.0020
OR (95% CI)
alcohol

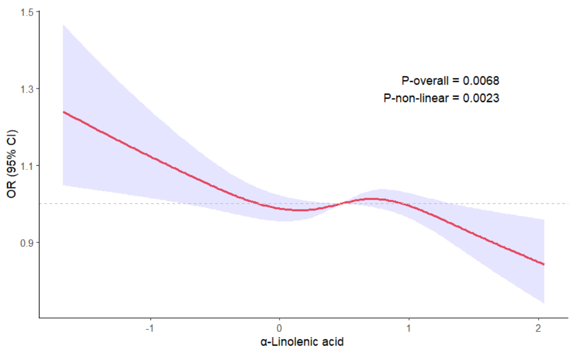

Figure S2. Restricted cubic spline plots of the association of liver fibrosis and nutrients
