# Supplementary material for: The association of dietary nutrients consumption with hepatic steatosis and fibrosis from NHANES 2017–2020
Source: Front Nutr. 2025 Jun 24;12:1510860. doi: 10.3389/fnut.2025.1510860 (PMC12234298; doi:10.3389/fnut.2025.1510860)
Supplement: Supplementary file 2 [file Table_1.doc]

**Supplementary Table 1.** Normal range of parameters

| **Parameter** | **Normal range** |
| --- | --- |
|  |  |
| Energy (kcal/day) | M ≥ 2400, F ≥ 1800 |
| Energy per weight (kcal/kg/day) | 25-35 |
| Protein (g/day) | M ≥ 56, F ≥ 46 |
| Protein per weight (g/kg/day) | < 65 years, ≥ 0.8 |
|  | ≥ 65 years, ≥ 1 |
| Carbohydrate (g/day) | ≥ 180 |
| Simple sugar (mg/day) | M < 36, F < 25 |
| Dietary fiber (g/1,000 kcal/day) | > 14 |
| Percentage of fat (%) | 20-35 |
| Percentage of saturated fat (%) | < 10 |
| Cholesterol (mg/day) | < 300 |
| Vitamin A, RAE (mcg/day) | M 900-3000 |
|  | F 700-3000 |
| Vitamin C (mg/day) | M 90-2000, F 75-2000 |
|  |  |
| Vitamin D (ug/mL) | ≥5 |
| Vitamin E (mg/day) | 15-1000 |
| Vitamin K (mcg/day) | M ≥ 120, F ≥ 90 |
| Thiamin (mg/day) | M ≥ 1.2, F ≥ 1.1 |
| Riboflavin (mg/day) | M ≥ 1.3, F ≥ 1.1 |
| Niacin (mg/day) | M 16-35, F 14-35 |
|  |  |
| Pyridoxine (mg/day) | ≤ 50 years, 1.3-100 |
|  | > 50 years, M 1.7-100 |
|  | > 50 years, F 1.5-100 |
| Folate (mcg/day) | 400-1000 |
| Cobalamin (mcg/day) | ≥ 2.4 |
| Calcium (mg/day) | M ≤ 70 years, 1000-2500 |
|  | M >70 years, 1200-2500 |
|  | F ≤50 years, 1000-2500 |
|  | F >50 years, 1200-2500 |
| Phosphorous (mg/day) | 700-4000 |
| Magnesium (mg/day) | M ≥ 420, F ≥ 320 |
| Iron (mg/day) | M 8-45 |
|  | F ≤ 50 years, 18-45 |
|  | F > 50 years, 8-45 |
| Zinc (mg/day) | M 11-40, F 8-40 |
| Copper (mg/day) | 0.9-10 |
| Sodium (mg/day) | ≤ 50 years, 1500-2300 |
|  | > 50-70 years, 1300-2300 |
|  | > 70 years, 1200-2300 |
| Potassium (mg/day) | ≥ 4700 |
| Selenium (mcg/day) | 55-400 |
| Caffeine (mg/day) | ≤ 400 |
| Alcohol (g/day) | M ≤ 28, F ≤ 14 |
| Linoleic acid (g/day) | ≤ 50 years, M ≥ 17, F ≥ 12 |
|  | > 50 years, M ≥ 14, F ≥ 11 |
| α-Linolenic acid (g/day) | M ≥ 1.6, F ≥ 1.1 |
| Fish oil (g/day) | ≥ 0.25 |
